# Invasive Fungal Infections after Liver Transplantation

**DOI:** 10.3390/jcm12093238

**Published:** 2023-04-30

**Authors:** Thomas Senoner, Robert Breitkopf, Benedikt Treml, Sasa Rajsic

**Affiliations:** Department of Anesthesia and Intensive Care Medicine, Medical University Innsbruck, 6020 Innsbruck, Austria

**Keywords:** invasive fungal infections, antifungal, antimycotic, candidiasis, aspergillosis, transplant, liver transplant

## Abstract

Invasive fungal infections represent a major challenge in patients who underwent organ transplantation. Overall, the most common fungal infections in these patients are candidiasis, followed by aspergillosis and cryptococcosis, except in lung transplant recipients, where aspergillosis is most common. Several risk factors have been identified, which increase the likelihood of an invasive fungal infection developing after transplantation. Liver transplant recipients constitute a high-risk category for invasive candidiasis and aspergillosis, and therefore targeted prophylaxis is favored in this patient population. Furthermore, a timely implemented therapy is crucial for achieving optimal outcomes in transplanted patients. In this article, we describe the epidemiology, risk factors, prophylaxis, and treatment strategies of the most common fungal infections in organ transplantation, with a focus on liver transplantation.

## 1. Introduction

Ever since the first liver transplant in 1963, orthotopic liver transplantation has become a life-saving standard of care treatment for end-stage liver diseases and malignancies [1]. Improvements in surgical techniques, organ preservation, immunosuppression, and critical care have pushed the one-year survival rates up to 90%, with a 10-year survival rate of more than 60% [2,3,4,5,6,7]. This resulted in a gradual increase in transplantation procedures, with 9236 liver transplantations performed in the USA in 2021, according to the Organ Procurement and Transplantation Network [8].

Due to immunosuppressive therapy, solid-organ transplant recipients are at increased risk of several complications, including rejection (mainly graft-versus-host disease) and infectious diseases (especially bacterial, viral, and fungal infections) [9]. Recent studies on more than 50,000 orthotopic liver transplant recipients identified the early postoperative period (first month after operation) as a period of increased risk of mortality [5,10,11]. Infections are identified as the most common cause of death during this period (39%) and dominated thereafter [5,12,13]. Due to iatrogenic immunosuppression, liver transplant recipients are prone to bacterial (abdominal collections, biliary tree, or catheters), and viral and fungal infections. Furthermore, this group of patients is prone to opportunistic infections, including multidrug-resistant organisms and invasive fungal infections (IFI) [14,15,16,17].

In October 2022, the WHO published the first fungal priority pathogens list, clearly highlighting solid organ transplant recipients as risk groups for IFIs [18]. Invasive fungal infections are one of the most important complications in this patient population, having an immense impact on the morbidity and mortality [19,20,21,22,23]. The IFI cumulative incidence in liver transplant recipients ranges from 5% to 42%, with a mortality rate from 25% to 80% [24,25,26,27,28,29].

**Figure 1:**
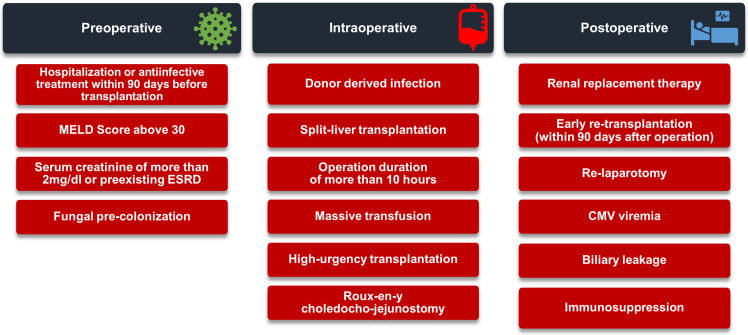
Risk factors for invasive fungal infections. Abbreviations: ESRD: end-stage renal disease, CMV: cytomegalovirus, MELD: Model for End-Stage Liver Disease Score, PRBC: packed red blood cells.

Multiple studies tried to identify the potential risk factors for IFIs, but due to the rather small sample size, retrospective nature of studies, and their methodology, the consensus is still missing. However, the potential risk factors can occur in three perioperative phases, with the potential for intervention at each stage. Depending on the time of occurrence, risk factors can be divided into the preoperative, intraoperative, and postoperative periods (Figure 1) [20,30,31,32,33,34].

Given the significant incidence of IFIs and their potential impact on patient outcomes, the implementation of an antimycotic prophylaxis is often being discussed. Recent guidelines recommend a risk-adjusted prophylaxis (targeted prophylaxis), with the choice of antifungal agent dependent on its antifungal spectrum against yeasts and/or molds, its toxicity, as well as potential drug interactions, pharmacokinetic metabolism, and tissue penetration [35]. Previous reports found a reduced incidence of IFIs when prophylaxis had been implemented, but without any influence on patient or graft survival [32,36,37,38,39,40,41]. The benefits of a universal antimycotic prophylaxis should be weighed against the possible dangers of resistance emergence and drug-related side effects. Instead, a targeted antimycotic prophylaxis directed only at patients with a high-risk profile is recommended [42]. However, systematized evidence and a consensus on the definition of high-risk patients, antimycotic agent selection, and the duration of prophylaxis for liver transplant recipients are still missing.

Therefore, in this article, we review the epidemiology, risk factors, diagnostic approach, therapeutic strategies, and the antifungal drugs most commonly used in clinical practice, with an outlook for the future development.

## 2. Epidemiology

Host and environmental factors mainly drive the epidemiology of fungal infections in solid-organ transplant recipients. Small bowel (11.6%) transplantation confers the highest risk, followed by lung (8.6%), liver (4.7%), heart (4%), pancreas (3.4%), and kidney (1.3%) transplants [29]. Host factors include prior colonization with the fungal agent, a breach in mucosal barriers, as well as several comorbid conditions such as diabetes, malnutrition, cirrhosis, or chronic kidney injury [43]. Environmental exposures to common fungi such as *Aspergillus* can lead to the chronic fungi carriage during the pre-transplantation stage, which can lead to an IFI once the patient is started on immunosuppressive therapy following transplantation [44]. The use of antifungal prophylaxis therapy has been shown to determine the type of fungal infection as well as its time of onset. Candidiasis is the most common IFI in transplanted patients, accounting for 50–60% of infections. *Candida* spp., primarily *Candida albicans*, are frequent colonizers of the gastrointestinal, respiratory, reproductive tract, and the skin. Thus, the majority of invasive candidiasis stems from endogenous sources–usually the skin or gut [29]. The second most common IFI is Aspergillosis, accounting for 20–25% of cases. However, in lung transplant recipients, it accounts for most cases of IFIs. Patients can get infected by means of the following two ways: either through reactivation of a previously quiescent process such as colonization or subclinical infection, or from a new infection in the post-transplantation phase, for example, via inhalation of a mold. *Cryptococcus* species (6–7%), the endemic fungi (5%), and many other rare and emerging mycoses cause the remaining infections [45].

The incidence of IFI in liver transplant recipients ranges from 4 to 40%. The incidence rises with time post-liver transplantation, with an overall rate at one year of 1.8%, increasing to 2.9% at 5 years and 5% at 10 years. Moreover, in liver transplant patients, *Candida* is the causative agent in the majority of cases (68–93%). Invasive *Aspergillus* infection occurs in 1–9% of patients following liver transplantation. The third most common IFI is *Cryptococcal* infection accounting for 0.5–5% of IFI [23].

Drug-resistant *Candida* strains represent a growing challenge in both prophylaxis and treatment of invasive candidiasis in patients with liver transplants [23]. Early diagnosis and rapid implementation of a specific therapy are crucial for a better outcome in these patients [46].

## 3. Risk Factors

Several factors that influence the risk of developing an IFI include the patient’s environmental exposure and/or colonization with pathogenic fungi, the type of surgery, total parenteral nutrition, use of antifungal prophylaxis, use of renal replacement therapy, including other factors such as broad-spectrum antibiotic use or transfusion of packed red blood cells, Table 1. Among surgeries, gastrointestinal procedures have been associated with the greatest risk of infection, followed by general abdominal surgery, elective surgeries, and major operations before ICU treatment.

Several studies have found an association between total parenteral nutrition and its duration with the risk of IFI development. Fungal colonization, especially in the digestive or respiratory tract, and renal replacement therapy, including its duration, have been associated with an increased risk of infection. Moreover, in orthotopic liver transplant recipients, the Model for End-Stage Liver Disease (MELD) score correlates with the increased risk of IFI development. However, the cut-off score and the range of the score, which would have an increased risk for IFI, differs between studies. For example, Saliba et al. reported on a MELD score of 20 or greater [20], while Utsumi et al. found that a score of 26 or greater increases this risk for IFI [47]. Thus, we could only conclude that the higher the MELD score, the greater the risk of IFI.

Multiple authors reported on the acute liver failure as an indication, the transplant operation time, split-liver, preexisting infections (i.e., cytomegalovirus—CMV), and choledochojejunostomy as established risk factors in the liver transplant recipients. Other reported risk factors include generalized infection/sepsis, as well as the need for mechanical ventilation, with the association of increased duration of ventilation with the risk of IFI. Moreover, Michalopoulos et al. showed that diabetes may increase the risk [48], whereas Paphitou et al. showed that diabetes did not play a significant role [49]. Finally, a number of other factors have been found as associated with an increased risk of IFI, such as increased Acute Physiology and Chronic Health Evaluation (APACHE) score, longer cardiopulmonary bypass time, acute renal failure, the use of broad-spectrum antibiotics, and insertion of peripheral or central venous catheters, Table 1.

**Table 1:** Risk factors associated with invasive fungal infections.

Risk Factors	Studies	OR (95% CI, *p*-Value)
**Surgery**		
Any surgery	Blumberg et al., 2001 [50]	7.3 (1–53.8, *p* = 0.05)
Multiple surgical procedures	McKinnon et al., 2001 [51]	Not reported, *p* ≤ 0.05
Repeated abdominal surgery	Nagao et al., 2016 [24]	5.2 (1–25.7, *p* = 0.046)
Surgery on ICU admission	León et al., 2006 [52]	2.71 (1.5–5.1, *p* < 001)
Elective surgery	Jordà-Marcos et al., 2007 [53]	2.75 (1.2–6.5, *p* = 0.02)
General abdominal surgery	Agvald-Öhman et al., 2008 [54]	60.7 (7.3-infinity, *p* = 0.001)
Gastrointestinal procedure	Chow et al., 2008 [55]	2.24 (1.5–3.4, *p* < 0.001) ^β^
Major pre-ICU operation	Chow et al., 2008 [55]	2.12 (1.1–4.0, *p* = 0.02) ^β^
Major operation during ICU stay	Chow et al., 2008 [55]	1.3, *p* = 0.04 ^α^
Choledochojejunostomy	Collins et al., 1994 [56] Viehmann et al., 2016 [30]	1.4, *p* = not reported 2.02 (0.45–9.07, *p* = 0.3) ^b^ 1.81 (0.99–3.32, *p* = 0.1) ^c^
Transplant operation time (h) 9–10.9 ≥11 Transplant operation time	Collins et al., 1994 [56] Viehmann et al., 2016 [30]	0.9, *p* = not reported 2.8, *p* = not reported not reported, *p* = 0.55 ^b^ not reported, *p* < 0.01 ^c^
Acute liver failure	Patel et al., 1996 [57] Shi et al., 2008 [58]	3.0 (1.1–7.9, *p* = 0.030) not reported, *p* = 0.002
**CMV infection**		
CMV infection	Badley et al., 1996 [59]	5.6 (2.5–12.7, p < 0.01)
Donor CMV+/recipient CMV-	George et al., 1997 [60]	4.8 (2.0–11.8, p = 0.001)
CMV infection	George et al., 1997 [60]	5.8 (1.8–18.4, p = 0.003)
CMV infection	Fortún et al., 2002 [61]	9.4 (1.21–89.6, p = 0.01)
**Total parenteral nutrition**		
	Borzotta & Beardsley, 1999 [62]	Not reported, *p* < 0.001
	Blumberg et al., 2001 [50]	3.8 (1.9–7.6, *p* < 0.001)
	León et al., 2006 [52] ^a^	2.5 (1.2–5.3, *p* < 0.001)
	Jordà-Marcos et al., 2007 [53] ^a^	3.9 (1.7–8.8, *p* = 0.001)
Total parenteral nutrition duration/days at risk	Chow et al., 2008 [55]	11 (5.5–21.7, *p* < 0.01) ^α^
** Fungal Colonization **		
* Candida * species corrected colonization index	Pittet et al., 1994 [63]	4.0 (2.2–7.5, *p* < 0.001)
Digestive focus	Ibàñez-Nolla et al., 2004 [64]	20.2 (6.1–67.0, *p* < 0.001)
Non- * Candida albicans * at screening	Ibàñez-Nolla et al., 2004 [64]	11.7 (1.9–70.6, *p* = 0.007)
Respiratory focus	Ibàñez-Nolla et al., 2004 [64]	6.6 (1.3–34.3, *p* = 0.026)
* Candida * colonization	León et al., 2006 [52] ^a^	3.0 (1.5–6.4, *p* < 0.001)
* Candida * colonization	Jordà-Marcos et al., 2007 [53] ^a^	4.1 (1.8–9.3, *p* = 0.001)
Colonization index ≥ 0.5	Agvald-Öhman et al., 2008 [54]	19.1 (2.4–435, *p* = 0.017)
** Renal replacement therapy **		
New-onset hemodialysis	Paphitou et al., 2005 [49]	5.4 (2.5–11.8, *p* = 0.029)
New-onset hemodialysis Hemofiltration	Nagao et al., 2016 [24] Jordà-Marcos et al., 2007 [53] ^a^	8.1 (2.4–27.6, *p* = 0.001) 2.0 (1.1–3.6, *p* = 0.032)
Hemodialysis duration/days at risk	Chow et al., 2008 [55]	3.8 (1.8–8.4, *p* < 0.001) ^α^
		6.2 (2.7–14.4, *p* < 0.001) ^β^
Infection/sepsis		
Hospital acquired	Michalopoulos et al., 2003 [48]	9.4 (2.5–48.3, *p* < 0.001)
Severe sepsis	León et al., 2006 [52] ^a^	7.7 (4.1–14.2, *p* < 0.001)
Enteric bacteremia	Chow et al., 2008 [55]	3.5 (1.4–8.6, *p* < 0.01) ^α^
		3.4 (1.4–8.4, *p* < 0.01) ^β^
** Mechanical ventilation **		
Mechanical ventilation after day 3	McKinnon et al., 2001 [51]	Not reported, *p* ≤ 0.05
Mechanical ventilation > 10 days	Michalopoulos et al., 2003 [48]	28.2 (3.6–119.5, *p* < 0.001)
** Diabetes **		
	Michalopoulos et al., 2003 [48]	2.4 (1.3–13.5, *p* < 0.01)
	Paphitou et al., 2005 [49]	2.8 (1.6–4.7, *p* = 0.053)
** APACHE score **		
APACHE II score	Pittet et al., 1994 [63]	1.0 (1.0–1.1, *p* = 0.007)
APACHE III score	Ibàñez-Nolla et al., 2004 [64]	1.0 (1.0–1.1, *p* = 0.004)
** Cardiopulmonary bypass time > 120 min **	Michalopoulos et al., 2003 [48]	8.1 (2.9–23.6, *p* < 0.01)
** Acute renal failure **	Blumberg et al., 2001 [50]	4.2 (2.1–8.3, *p* < 0.001)
** MELD score **		
MELD score	Alexander et al., 2006 [65]	1.0 (1.0–1.1, *p* = 0.003)
MELD score 20–30	Saliba et al., 2013 [20]	2.1 (1.2–3.7, *p* = 0.012)
MELD score ≥ 30	Saliba et al., 2013 [20]	3.1 (1.6–6.0, *p* < 0.001)
MELD score ≥ 26	Utsumi et al., 2019 [47]	16.0 (3.0–118.3, *p* = 0.001)
** Broad-spectrum antibiotics **	Paphitou et al., 2005 [49]	3.0 (1.8–5.0, *p* = 0.028)
** Packed red blood cell transfusion **		
	Chow et al., 2008 [55]	2.0 (1.0–4.0, *p* = 0.06) ^α^
		2.7 (1.3–5.6, *p* < 0.01) ^β^
** Antifungal medication **		
Antifungal medication	Blumberg et al., 2001 [50]	0.3 (0.1–0.6, *p* < 0.001)
Prior use of antifungal therapy Central venous catheters	Kim et al., 2019 [66] McKinnon et al., 2001 [51]	13.6 (3.0–61.0, *p* < 0.001) Not reported, *p* ≤ 0.05
Diarrhea	McKinnon et al., 2001 [51]	Not reported, *p* ≤ 0.05
Peripheral catheter use	McKinnon et al., 2001 [51]	Not reported, *p* ≤ 0.05

APACHE: Acute Physiology and Chronic Health Evaluation; CMV: cytomegalovirus; CPB: cardiopulmonary bypass; ICU: intensive care unit; MELD: Model for End-Stage Liver Disease; ^α^: OR for outcomes in *Candida albicans*; ^β^: OR for outcomes in *Candida non-albicans*; ^a^: data combined from both articles from the EPCAN Study; ^b^: for superficial invasive fungal infections; ^c^: for deep invasive fungal infections.

Given the recent severe acute respiratory syndrome (SARS) coronavirus disease (COVID) 2019 pandemic, its implication in patients undergoing solid organ transplantation might be of clinical relevance. A retrospective study analyzed data of solid organ transplant recipients hospitalized with COVID-19, with 59% having a kidney transplantation, 17% a lung, 11% each having a heart or liver, and 2% a small bowel transplantation. Among these patients, 8% developed IFI within 90 days of COVID-19. The 90-day mortality after COVID-19 diagnosis was higher for patients with IFI (57% vs. 20%) [67]. Given the short time span that passed since the emergence of COVID-19, no recommendation can be made regarding the treatment of IFI in patients with COVID-19 infection.

## 4. Diagnostic Approach: Scores and Biomarkers

According to the consensus definition of the European Organization for Research and Treatment of Cancer and the Mycoses Study Group Education and Research Consortium (EORTC/MSGERC), the diagnosis of IFI can be made using various tools, such as microscopic analysis, cultures (sterile material or blood) and tissue nucleic acid amplification by polymerase chain reaction, or serology in the case of cryptococcal disease [68].

In addition to the criteria for a “proven” infection, the categories “probable” and “possible” are also suggested for immunocompromised depending on the level of probability (except for endemic mycoses). Criteria for the proven disease vary depending on the fungus (molds, yeasts, pneumocystis, endemic mycoses), but require the detection of the fungal organism through histopathological or culture methods from sterile sites. In case of probable disease, the definition includes features such as host factors (history of neutropenia, receipt of an allogeneic stem cell transplant, prolonged use of corticosteroids, immunosuppression therapy, inherent immunodeficiency), clinical and radiological signs of tracheobronchitis, sinonasal or central nervous system infection, as well as the type of mycological evidence (e.g., (1-3)-beta-D-glucan (BDG) ≥80 ng/L detected in at least two consecutive serum samples provided that other etiologies have been excluded in case of candidiasis, or recovery of any mold from sputum, bronchoalveolar lavage, bronchial brush or aspirate, Galactomannan antigen detected in plasma, serum, bronchoalveolar lavage or cerebrospinal fluid in case of *Aspergillosis*). A probable diagnosis is attributed to a patient when the parameters of host factors, clinical signs, and mycological evidence through molecular methodologies or serological tests are present. Possible infections are diagnosed if host factors and clinical signs strongly indicate an IFI, but the mycological evidence parameter is still missing [69].

Finally, it has also been suggested to differentiate between definitions of invasive fungal disease required for clinical research from those that influence clinical practice [68]. Moreover, the definition of breakthrough infections is another important topic. A breakthrough infection is defined as any IFI occurring during exposure to an antifungal drug, including fungi outside the spectrum of activity of an antifungal [70].

Candidemia was defined as the isolation of *Candida* spp. from at least one blood culture. Other means of diagnosing invasive candidiasis (deep-seated candidiasis) is by culture, staining, and/or histopathology of samples acquired by biopsy or aspiration of involved tissue. Blood cultures have their own flaws, with a reported sensitivity for detecting *Candida* spp. of 50–75%. Thus, the guidelines for the diagnosis and management of Candida infections by the European Society of Clinical Microbiology and Infectious Diseases (ESCMID) recommend alternative techniques [71]. In serum samples, the detection of mannan and anti-mannan antibodies is considered to be a method for the specific detection of *Candida* spp. This method has a sensitivity of 80% and a specificity of 85% and has also a very high negative predictive value (>85%), making it useful to rule out infection.

Due to the limitations of culture-based techniques, culture-independent diagnostic tests have been developed. The three most robust diagnostic tests are BDG, Candida polymerase chain reaction (PCR), and T2 Candida assays in serum. The T2 Candida magnetic resonance assay is a novel technique capable of directly detecting Candida cells in whole blood. Candida cells are lysed by mechanical bead beating, Candida DNA is then amplified with a thermostable DNA polymerase and bundled into magnetic nanoparticles, which then can be detected in magnetic resonance [72]. The BDG test is considered to be a panfungal diagnostic method and thus is not specific for Candida because it is present in many fungal species [71]. PCR can specifically target *Candida* spp. and thus offers advantages over BDG, with a high sensitivity and specificity (95% and 92%, respectively) being reported. T2 Candida detects the 5 most common *Candida* spp. within whole blood by an automated process in which amplified DNA targets are detected by T2 magnetic resonance. Moreover, this test has a high sensitivity and specificity for candidemia (89–91% and 98%, respectively) [73]. A score has been developed for the detection of invasive candidemia in non-neutropenic critically ill patients, namely, the “Candida score”. The score was evaluated in a multicenter surveillance study including 1699 ICU patients, and a “Candida score” >2.5 was found to accurately select patients who would benefit from early antifungal treatment [52]. To our knowledge, no score has been developed for the detection of invasive fungal diseases in solid organ transplant patients.

For the diagnosis of Aspergillosis, galactomannan, BDG, and PCR are standard diagnostic tests used in clinical practice. Galactomannan can be detected in urine, bronchoalveolar lavage fluid, cerebrospinal fluid, and other specimens with enzyme immunoassay. The presence of galactomannan in the circulation correlates with the invasive growth of *Aspergillus* spp. through the pulmonary capillaries and invasion of blood vessels has been correlated with fungal burden and galactomannan production. Thus, the performance of the test depends upon disease burden, with patients with hematologic malignancies and allogeneic hematopoietic stem cell transplant recipients having a higher burden of disease compared with solid organ transplant patients; consequently, the performance of the test is relatively poor in the latter group [73].

Among the EORTC/MSG criteria for proven invasive fungal disease, tomographic signs of lower respiratory tract infections such as dense, well-circumscribed lesions(s) with or without a halo sign, or delayed findings such as an air-crescent sign (crescent-shaped collection of air surrounding an infarcted sequester) or a cavity stay are the golden standard of radiological diagnostics [74]. Thin-section computed tomography (CT) evaluation is required within 12–24 h of symptom onset at an optimized dose. Although contrast media are not obligatory, CT-angiography may provide additional important information about direct peripheral vascular occlusion in lesions with a large diameter and not localized in peripheral lung parenchyma with high sensitivity and negative predictive value for invasive pulmonary aspergillosis [75,76]. As an alternative magnetic resonance imaging (MRI) with T2-weighted turbo-spin-echo sequences exhibits sensitivity and specificity approaching that of CT for the diagnosis of invasive aspergillosis (IA) [77]. CT of the nasal fossae and paranasal sinuses may allow an early diagnosis of sinunasal fungal infections, such as invasive aspergillosis or mucormycosis, with an optimal assessment of osseous erosion. Nevertheless, MRI has higher sensitivity and specificity and better visualization of cerebral lesions [78]. Cerebral affections of IFI are, in general, diagnosed by CT and MRI, and CT is ideal for assessing bone involvement and has to include a non-enhanced series to exclude bleeding. An ordinary CT requires an additional MRI, which has better sensitivity for small lesions that may be undetected by CT [79].

## 5. Clinical Manifestations and Infection Sites

Although IFI in transplanted patients can affect virtually any organ, several predilected sites of infection have been identified, which vary depending on the mold. Invasive candidiasis most commonly involves the bloodstream and/or the abdomen. Candidiasis in the blood can arise from the translocation of organisms across damaged intestinal mucosa or from an infected central venous catheter [44]. Candidemia accounts for the majority of IFI in liver transplant patients, followed by intraabdominal candidiasis (e.g., peritoneal, perinephric, and biliary infections). In a retrospective, multicenter study, among intraabdominal infections, peritonitis and abdominal abscesses were the most common types (38.9% in both cases), followed by biliary tract infections (16.7%). Invasive Candida infections tend to occur early after transplantation, with about 34% and 46% of cases occurring during the first month and within three months, respectively. Early infection (within the first three months) has been associated with an increased likelihood of being hospitalized in an intensive care unit and acute kidney injury development [80]. Biliomas are a potentially devastating complication of liver transplantation since Candida has an affinity for growth in the biliary tract and bile extracts, which significantly decreases its antifungal susceptibility [81]. Candida can be identified in approximately 25% of such infections. Finally, biliomas are associated with high mortality and the need for re-transplantation [82]. Airway or lung infection (as opposed to colonization) with Candida has become rare thanks to antifungal prophylaxis. *Candida* spp. are frequently found in respiratory samples from lung transplant recipients or donors, even though such findings are rarely clinically relevant [83]. Candiduria is common, with an estimated incidence of about 4% in kidney transplant recipients. Most patients are asymptomatic, and antifungal treatment had no impact on candiduria clearance, as reported in a single-center retrospective study of 1223 kidney transplant patients [84].

Infection with *Aspergillus* spp. almost always involves the respiratory tract and/or sinuses. Transmission of Aspergillus at the time of transplantation has been documented and occurs either directly from an infected organ or due to contamination of organ preservation fluid by airborne spores [85,86]. Unusual sites of infection, such as the urinary tract, graft anastomosis, and heart valve(s) are suggestive of donor-derived infection. Lung transplant recipients carry the highest risk of aspergillosis infection [29]. As compared to Candida infections, infections with *Aspergillus* spp. tend to occur later after transplantation. Even though earlier epidemiologic studies reported on the occurrence of invasive aspergillosis within 17 days after transplantation, more recent cohort studies indicate that the median time to infection is nowadays >100 days. Disseminated disease with IFI is common in liver transplant recipients (55%) and is associated with a high mortality rate (64%) [87].

Donor-derived infections are rare; however, they can be associated with serious complications in transplant recipients. Most cases of donor-derived candidiasis have been reported in kidney transplant patients, where contaminated preservation fluid is thought to be the most common source of infection. Transmissions from donors with candidemia have also been described. Vascular complications such as mycotic aneurysms and anastomotic ruptures represent the most serious manifestations of these infections [88]. Vascular complications have been associated with (massive) bleeding, graft loss, and increased mortality [89]. Aspergillus is a less common organism in donor-derived fungal infections [88].

## 6. Therapeutic Strategies: Prophylactic, Empiric, Preemptive

Antifungal prophylaxis in solid organ transplant recipients has been established based on the rising incidence of life-threatening IFI, the diagnostic difficulties in the early stage of infection, and the evidence that the treatment outcome is poor if there is a delay in the therapy initiation. Therefore, the following three strategies have been described to prevent fungal infections: universal, targeted, and preemptive prophylaxis. Universal prophylaxis refers to the administration of antifungal agents to all patients before the isolation of a fungal pathogen in the postoperative period. Targeted prophylaxis refers to the administration of antifungal agents, such as with universal prophylaxis, but only to high-risk patients, and preemptive treatment refers to administering antifungal agents in the postoperative period to patients with only fungal colonization and without evidence of invasive fungal disease [90].

Antifungal prophylaxis in liver transplantation has been shown to significantly reduce the risk for proven IFI (OR 0.37, 95% CI 0.19–0.72, *p* = 0.003). Furthermore, it may also reduce the incidence of suspected and proven fungal infections, superficial fungal infections, and fungal colonization. With the use of systematic prophylaxis, mortality due to fungal infection is significantly reduced (OR 0.32, 95% CI 0.10–0.83, *p* = 0.02), although this did not translate into a reduction in all-cause mortality (OR 0.87, 95% CI 0.54–1.39, *p* = 0.55). Of notice, antifungal prophylaxis has not been shown to reduce the incidence of Aspergillus IFI [42]. Given the high morbidity and mortality rates associated with IFI, most transplant centers employ a certain strategy of antifungal prophylaxis. In lung transplant recipients, either universal prophylaxis or preemptive therapy is recommended, whereas targeted prophylaxis is favored in liver and heart transplant recipients [87]. Liver transplant recipients constitute a high-risk category for invasive candidiasis and aspergillosis. In the absence of antifungal prophylaxis, IFI occur in 36% of transplant patients [91]. Therefore, targeted prophylaxis with antifungal agents active against *Candida* spp. and *Aspergillus* spp. is recommended. In clinical practice, fluconazole is the most commonly used first-line drug. A recent meta-analysis showed similar efficacy with the lipid formulation of amphotericin B in high-risk liver transplant recipients [42]. However, due to the widespread use of fluconazole, patients have an increased risk of developing an infection with a fluconazole-resistant organism [92]. In liver transplant patients, targeted prophylaxis with anidulafungin, micafungin or caspofungin in a standard dose, or voriconazole is recommended against invasive aspergillosis. Moreover, targeted prophylaxis with a lipid formulation of amphotericin B may be considered. The optimal duration of targeted prophylaxis has not been established yet. In most centers, prophylaxis is being given over a time span of 14–21 days [87].

## 7. Antifungal Pharmacotherapy: Substances, Susceptibility, Therapeutic Drug Monitoring (TDM)

Fungal pathogens are prone to long-lasting deep-seated infections, resulting in suboptimal antifungal pharmacokinetics with a lack of antifungal activity during treatment. Currently, polyenes, flucytosine, azoles, and echinocandins are the only major classes of antifungal agents existing.

Within the first group, polyenes interact with ergosterol-containing fungal membranes by interacting with cholesterol-containing membranes and damaging the host cells. Flucytosine presents a pyrimidine analog, while azoles primarily block ergosterol synthesis by inhibiting lanosterol 14α-demethylase (Erg11). Finally, echinocandins block BDG synthase. Aside from isavuconazole, which was approved in 2015 to treat aspergillosis and mucormycosis, no new class of antifungal drugs has been implemented in more than a decade.

Given the above, the development of secondary resistance is often associated with a high rate of treatment failure [93]. The mechanisms of resistance vary among the antifungal agents. Polyene resistance is rarely acquired but occurs primarily in fungal species whose ergosterol membranes are not or are only slightly attacked by polyenes. In addition, polyenes can lose their fungicidal activity with prolonged exposure [94]. In contrast, azoles have multiple mechanisms for the development of both primary and acquired resistance (e.g., mutations in the gene encoding lanosterol-14α-demethylase or enhanced/induced efflux pumps) [95]. Echinocandin resistance is based on the development of mutations in FKS1, which encodes the BDG enzyme involved in cell wall synthesis [96]. Recently, mutations in MSH1, a mismatch repair gene involved in resistance development, have also been observed [97].

Therefore, a timely implemented therapy is crucial for achieving optimal outcomes in transplanted patients. The list of common antifungal agents, their spectrum of activity, major toxicities, and drug interactions is presented in Table 2.

### 7.1. Invasive Candidiasis

Therapy of invasive candidiasis in transplant patients does not differ from the treatment of non-neutropenic patients, even though some aspects related to drug-drug interactions and potential toxicities associated with azoles should be considered. Certain antifungals should not be used in transplanted patients, as, for example, amphotericin B deoxycholate should not be used due to its nephrotoxicity, especially in patients receiving calcineurin inhibitors. Furthermore, all the azoles interact with calcineurin inhibitors via the cytochrome P450 enzymes, and thus determination of plasma levels of both azoles and immunosuppressive agents is recommended. Echinocandins have fewer side effects, less nephrotoxicity, and fewer drug-drug interactions compared with other antifungal agents and have shown high success rates for the treatment of invasive candidiasis [43]. The Infectious Diseases Society of America guidelines from 2016 recommend an echinocandin as initial therapy for candidemia in non-neutropenic patients, but there are doubts as to whether this recommendation can also be applied to intra-abdominal infections [92].

The low echinocandin susceptibility of *C. parapsilosis*, as well as recent reports about rising echinocandin resistance rates due to point mutations in the FKS1 and FKS2 genes (FK506 sensitivity genes, also referred to as GSC1; orf19.292 and GSL2; orf19.3269) after echinocandin exposure among *C. glabrata*—especially in the clinical setting of an intraabdominal candidiasis—have become a matter of concern [98,99,100]. Pathophysiological changes of pharmacokinetics in critically ill patients due to sepsis, hypoalbuminemia, capillary leakage, or altered renal function may prevent achieving target concentration in both the plasma and primary infection sites. Moderate penetration of echinocandins into the peritoneal fluid in patients with intra-abdominal candidiasis may therefore result in secondary echinocandin resistance among initially echinocandin-sensitive strains of *C. glabrata*, which colonize and survive in a “protected” reservoir [101].

Fluconazole is an acceptable alternative to an echinocandin in selected patients, including those who are not critically ill and who are considered unlikely to have a fluconazole-resistant *Candida* spp. Lipid formulation amphotericin B is a reasonable alternative if there is intolerance, limited availability, or resistance to other antifungal agents [92]. Additionally, central venous catheters (CVCs) should be removed as early as possible in the course of candidemia when the source is presumed to be the CVC, and the catheter can be removed safely. According to the most actual recommendations, antifungal therapy should be continued for two weeks after documented clearance of *Candida* spp. from the bloodstream and resolution of symptoms attributable to candidemia [92].

### 7.2. Invasive Aspergillosis

The azoles, the polyenes, and the echinocandins are three main classes of antifungal agents in clinical use for the treatment of invasive aspergillosis. Voriconazole is the drug of choice, with isavuconazole and lipid formulation of amphotericin B regarded as alternative agents [87]. Due to its penetration into the central nervous system (CNS), voriconazole has improved the prognosis of patients with invasive aspergillosis when the CNS is affected [43]. Liposomal amphotericin B is usually the drug of choice in patients with liver insufficiency. Posaconazole is mainly used in the treatment of cases that are refractory or intolerant to other first-line antifungal agents. The echinocandins are typically used alone or in combination for salvage therapy. In a randomized controlled trial, voriconazole was shown to be superior to amphotericin B deoxycholate in terms of survival [87].

## 8. Future Development and Outlook

Due to the demographic change and the over-aging structure of our society, as well as recent HIV- and SARS-CoV-2 (epi-)pandemics, the widespread use of fungicides in agriculture and the dissemination of medical interventions, including modern oncologic chemotherapies, new monoclonal antibodies with immunological properties, immunosuppressive drugs and broad-spectrum antimicrobials, the burden of fungal diseases is notably rising worldwide.

The global incidence of invasive candidiasis has been estimated to be 750,000, including 60,000–100,000 cases of intra-abdominal candidiasis, in the case of invasive aspergillosis, more than 300,000 per year [102]. Although Austria is still one of the countries with the lowest incidences of candidemia (206 cases, 2.1 per 100,000), the numbers continue to rise with a worrying increase of reduced azole-susceptible non-albicans *Candida* spp., such as *C. glabrata* and *C. krusei*, as well as reduced echinocandin susceptibility of *C. parapsilosis* and *C. guilliermondii* [103]. Recent reports show a rising percentage of co-resistance to both azoles and echinocandins in *C. glabrata* isolates [104]. Since its identification in 2009, *C. auris,* with its extensive innate and acquired resistance to antifungal drugs and widely used hospital disinfectants, has become a globally emerging infection [105]. Among *Aspergillus* spp., *A. fumigatus* is the most frequently isolated mold, but in Austria, rising numbers of *A. terreus* have been noticed to be of serious concern due to its amphotericin B resistance [106].

Moreover, the diagnosis of systemic fungal infections remains problematic. Fungal cultivation, as the golden standard for diagnosis, was for a long time limited by its low sensitivity and long growth time, but nowadays, the causative fungal pathogens can be identified quickly and accurately through new technologies such as matrix-assisted laser desorption/ionization time-of-flight mass spectrometry (MALDI-TOF), fluorescence in situ hybridization (FISH), PCR and T2-MRI technology, and serological biomarkers [107].

Concerning the therapeutic options, the currently available offer is limited. To achieve better outcomes, drugs must kill yeasts or molds rapidly and completely. Current treatments take too long and thus reduce the immediate fungicidal activity and therapy compliance. Drug-related toxicity and the emergence of resistance limit treatment and contribute to poor outcomes since affected patients often show high frailty with limited tolerance to additional organ toxicity or drug interactions. New strategies are urgently needed. Mycoviruses that selectively infect fungi, tetrazoles, the echinocandin rezafungin, or the glucan synthase inhibitor Ibrexafungerp are possible promising hopes for the future [108,109,110,111,112,113]. New mechanisms of action are also being considered, for example, inhibiting the inositol acyltransferase Gwt1 or the dihydroorotate dehydrogenase [114,115]. Aureobasidin A, a cyclic depsipeptide inhibiting fungal sphingolipid biosynthesis, or the novel arylamidine T-2307, which selectively disrupts yeast mitochondrial function by inhibiting respiratory chain complexes, are further possible future antifungal agents [116,117]. Novel anti-virulence approaches, such as the inhibition of biofilm formation (in contrast to the inhibition of fungal growth), reduce the selective pressure as they are less likely to induce antifungal resistance [118]. In his review, the author John R. Perfect focuses on promising pathways and specific targets of future antifungal therapies [119].

The antifungal agents currently used in clinical practice have certain limitations owing to their toxicities and due to emerging resistance to these agents. The development and discovery of novel antifungal agents could alleviate this problem. Furthermore, judicious implementation of prophylactic strategies, i.e., the right prophylactic strategy for the right patient, is crucial to minimize the emergence of resistant strains. The duration of prophylaxis and the best antifungal agent for prophylaxis is still a matter of debate, and more research is needed to clarify these issues.

Due to the rising resistance to antifungal agents, the implementation of a biomarker-based surveillance has been suggested by the ESCMID guidelines [120]. A surveillance approach could include regular, twice-weekly biomarker monitoring coupled with clinical assessment. Due to the high negative predictive value, a negative surveillance biomarker result could reassure the clinician that despite the presence of prolonged fever, empirical antifungal therapy is not required and should be discontinued in patients where the risk of IFI has been deemed to be low [121]. Indeed, prospective studies have been conducted using surveillance biomarkers, and they could demonstrate a substantial reduction in empirical antifungal use [122,123].

## 9. Conclusions

Invasive antifungal infections in transplanted patients are likely to increase in the future, partially owing to the increased number of patients being transplanted worldwide. Toxicities of currently available antifungal agents and emerging resistance to these agents are limiting the treatment possibilities in these complex, multimorbid patients. Thanks to the widespread use of prophylactic agents in transplanted patients, morbidity and mortality could be reduced in the past years. Clinicians treating transplanted patients need a thorough knowledge of the most common molds, their clinical manifestations and the site of infection, the varying prophylactic strategies in different solid organ transplantations, and the best therapeutic method for these patients. With the increasing knowledge about invasive antifungal infections, it is possible nowadays to implement a patient-centered and individualized therapy for transplanted patients.

## Figures and Tables

**Table 2:** Comparison of the spectrum of activity, major toxicities, and drug interactions of systemic antifungal agents in liver transplant recipients.

Agent	Spectrum of Activity	Major Toxicity	Interaction with Immunosuppressants	Comments
***Azoles***				
Fluconazole	Candidiasis Cryptococcosis Coccidioidomycosis	Hepatotoxicity, QT interval prolongation	Increases levels of CNI and MTI	Oral and i.v. formulations; currently insufficient evidence to support the routine use of TDM
Isavuconazole	Aspergillosis Mucormycosis	Hepatotoxicity	Increases levels of CNI and MTI	Oral and i.v. formulations; currently insufficient evidence to support the routine use of TDM
Itraconazole	CandidiasisEndemic mycosisAspergillosis	Hepatotoxicity, QT interval prolongation, negative inotropic effect	Increases levels of CNI and MTI	Oral formulation only; syrup solution is better absorbed compared to tablets; higher costs; TDM may be helpful; target trough level is >0.5–1 mg/L
Posaconazole	CandidiasisAspergillosisEndemic fungiRare and emerging molds	GI intolerance, hepatotoxicity, QT interval prolongation	Increases levels of CNI and MTI	Oral formulation (syrup) only; delay of several days to achieve steady state levels; TDM may be helpful Treatment target trough level >1 mg/L (preferably >1.25 mg/L)
Voriconazole	CandidiasisAspergillosisRare and emerging molds	Hepatotoxicity, QT interval prolongation, psychosis, visual changes, dermatitis	Increases levels of CNI and MTI, caution with sirolimus	Oral and i.v. formulations; TDM may be helpful; treatment target trough level is >1 mg/L; level of 1–5.5 mg/L is considered adequate for most patients; higher target (e.g., 2–6 mg/L) should be used if there is disease with a poor prognosis (e.g., CNS infection, bulky disease, multifocal infection)
***Polyenes***				
Deoxycholate Amphotericin B (AmB)	Broad range of yeasts and molds	Renal, electrolyte and infusion-related toxicities	Increased nephrotoxicity with CNI	Aerosol and i.v. formulation; rarely used in SOT due to nephrotoxicity; currently insufficient evidence to support the routine use of TDM
Lipid formulations of AmB	Broad range of yeasts and molds	Renal, electrolyte and infusion-related toxicities, but less than deoxycholate	Increased nephrotoxicity with CNI, but less than deoxycholate	Aerosol and i.v. formulation; currently insufficient evidence to support the routine use of TDM
***Echinocandins***				
Anidulafungin	CandidiasisAspergillosis	Rather rare, rash, hepatotoxicity	Cyclosporine increases anidulafungin level	Only i.v. formulation; currently insufficient evidence to support the routine use of TDM
Caspofungin	CandidiasisAspergillosis	Rather rare, rash, hepatotoxicity	Decreased tacrolimus levelCyclosporine increases caspofungin level	Only i.v. formulation; currently insufficient evidence to support the routine use of TDM
Micafungin	CandidiasisAspergillosis	Rather rare, rash, hepatotoxicity	Increased cyclosporine and sirolimus levels	Only i.v. formulation; causes liver tumors in rats (black-box warning in Europe); currently insufficient evidence to support the routine use of TDM
***Others***				
Flucytosine	Cryptococcosis (in combination with AmB)	Bone marrow and liver toxicity	Increased myelosuppression with sirolimus and mycophenolate mofetil	Oral formulation only; drug levels are proportional to dose and renal dysfunction; TDM may be helpful

Abbreviations: AmB: amphotericin B; CNI: calcineurin inhibitors (e.g., tacrolimus and cyclosporine); GI: gastrointestinal; i.v.: intravenous; MTI: mTOR inhibitors (e.g., sirolimus and everolimus); SOT: solid organ transplant; CNS: central nervous system; TDM: therapeutic drug monitoring.

## Data Availability

Not applicable.
